# Temporal Modeling of Amyloid and Tau Trajectories in Alzheimer's Disease Using PET and Plasma Biomarkers

**DOI:** 10.1002/ana.78194

**Published:** 2026-03-15

**Authors:** Christopher A. Brown, Katheryn A.Q. Cousins, Magdalena Korecka, Emily McGrew, Alice Chen‐Plotkin, John A. Detre, Corey T. McMillan, Edward B. Lee, Sandhitsu R. Das, Dawn Mechanic‐Hamilton, Paul A. Yushkevich, Ilya M. Nasrallah, Leslie M. Shaw, for the Alzheimer's Disease Neuroimaging Initiative, David A. Wolk

**Affiliations:** ^1^ Department of Neurology University of Pennsylvania Philadelphia PA USA; ^2^ Department of Pathology and Laboratory Medicine University of Pennsylvania Philadelphia PA USA; ^3^ Department of Radiology University of Pennsylvania Philadelphia PA USA

## Abstract

**Objective:**

This study aimed to compare positron emission tomography (PET) and plasma‐based temporal modeling of amyloid and tau biomarkers in Alzheimer's disease.

**Methods:**

Longitudinal amyloid PET (n = 1,097, mean age ± SD = 72.5 ± 7.38 year, 51.4% male), ^18^F‐flortaucipir tau‐PET (n = 230, 74.3 ± 7.18 year, 52.2% female), and Fujirebio Lumipulse plasma p‐tau_217_ (n = 752, 72.8 ± 6.93 year, 51.3% male) from the Alzheimer's Disease Neuroimaging Initiative (ADNI) and University of Pennsylvania Alzheimer's Disease Research Center (Penn ADRC) were used to generate biomarker trajectory models using sampled‐iterative Local approximation (SILA). SILA models using plasma p‐tau_217_ were compared to amyloid and tau PET‐based models to estimate amyloid and tau onset, and factors influencing tau onset and time from tau onset to dementia were evaluated for PET and plasma models.

**Results:**

Plasma and PET models generated similar results for estimated amyloid and tau onset, with stronger model agreement for tau (*r* = 0.88[0.86, 0.89], *t* = 57.4, *p* < 0.001) than amyloid (*r* = 0.75[0.72, 0.77], *t* = 37.4, *p* < 0.001) onset. Accuracy of estimated onset compared to actual onset was high within modality (mean absolute error [MAE] ≤ 2.03) with slightly greater error (MAE 3.09–3.42) when comparing across modalities (ie, plasma to PET). For both plasma and PET, earlier tau onset was associated with younger amyloid onset, female sex, and ≥1 apolipoprotein (ApoE) ε4 allele. Earlier dementia onset after tau was associated with later tau onset for both plasma and PET, while male sex was associated with shorter tau to dementia gap in plasma models.

**Interpretation:**

Temporal modeling of plasma biomarkers provides comparable information to PET‐based models, particularly for tau onset age, and can serve as a widely accessible tool for clinical assessment of biological disease severity. ANN NEUROL 2026;99:1438–1451

Alzheimer's disease (AD) is characterized by the presence of amyloid‐β (Aβ) plaques followed by the development of tau neurofibrillary tangles, neurodegeneration, and cognitive decline.^1^, ^2^, ^3^ While the sequence of these events within AD have been well‐characterized, there is significant heterogeneity in the specific timing of each of these events.^4^, ^5^, ^6^ Moreover, the presence of co‐pathology can alter the timing of neurodegeneration and cognitive impairment relative to amyloid and tau onset.^6^, ^7^, ^8^, ^9^ Understanding the factors that influence the timing of biomarker changes and alterations to the sequence of these events is particularly important for assessing response to interventions in clinical trials and clinical practice.

Recently developed temporal modeling methods of AD biomarkers allow for estimates of the time of amyloid and tau onset.^10^ To date, this literature has focused on amyloid and tau positron emission tomography (PET) to model estimated amyloid and tau onset age.^11^, ^12^ After determining these estimated ages, it is possible to anchor longitudinal and cross‐sectional analyses to these estimated onset ages, rather than the arbitrary times at which biomarkers are collected. Using this approach, studies have demonstrated the impact of apolipoprotein E (ApoE) status, age, sex, and education on progression to dementia after estimated amyloid onset, as well as factors influencing estimated tau onset age.^10^, ^11^, ^12^ By providing estimated biological disease onset and duration, these methods may be particularly useful in the clinic to improve prognostic predictions, where biomarkers are often not collected until after the onset of symptoms and longitudinal collection is limited by costs and geographic availability. While these studies highlight the value of temporal modeling, they have primarily been applied to PET biomarkers, which are less available in clinical settings outside of resource‐rich areas.

Over the past several years, significant advances have been made in the accuracy and robustness of plasma biomarkers of AD pathology, especially plasma phosphorylated tau 217 (p‐tau_217_) which is strongly associated with both tau and amyloid pathology. This has led to the recent United States Food and Drug Administration (FDA)‐approval of the Fujirebio Lumipulse p‐tau_217_/Aβ_42_ ratio as the first blood‐based biomarker for detection of Aβ positivity (Aβ+). As these measures become more ubiquitous in both research and clinical practice, application of temporal modeling approaches to plasma biomarkers could allow for more widespread and direct translation to clinical practice. Here, we used longitudinal data from the Alzheimer's Disease Neuroimaging Initiative (ADNI) and University of Pennsylvania Alzheimer's Disease Research Center (Penn ADRC) cohort to generate temporal models of PET and plasma biomarker trajectories using the previously validated sampled‐iterative local approximation (SILA) method.^10^ We examined the overlap between PET and plasma models, as well as investigated the accuracy of these models in predicting true Aβ and tau positivity in a subset of individuals who converted during longitudinal follow‐up. Finally, we examined factors contributing to estimated tau onset age and time from tau onset to dementia onset for both PET‐ and plasma‐based models.

## Materials and Methods

### Participants

Individuals were selected from ADNI and Penn ADRC based on availability of: (1) amyloid PET Centiloid (CL) quantification (ADNI only), (2) ^18^F‐flortaucipir tau PET with Tau‐MaX calculation,^13^ (3) or Fujirebio Lumipulse p‐tau_217_. The inclusion and exclusion criteria of these cohorts have been previously published,^14^, ^15^ and written informed consent was obtained from all participants under protocols approved by the local Institutional Review Boards (IRBs) in accordance with the Declaration of Helsinki. For both ADNI and Penn ADRC, demographic information and longitudinal clinical diagnoses are collected using procedures described previously.^14^, ^15^

A subset of data used in the preparation of this article were obtained from the ADNI database (adni.loni.usc.edu). The ADNI was launched in 2003 as a public‐private partnership, led by Principal Investigator Michael W. Weiner, MD. The primary goal of ADNI has been to test whether serial magnetic resonance imaging (MRI), PET, other biological markers, and clinical and neuropsychological assessment can be combined to measure the progression of mild cognitive impairment (MCI) and early Alzheimer's disease (AD).

### PET Acquisition and Analysis

Amyloid PET was collected 50 to 70 minutes or 90 to 110 minutes after injection using ^18^F‐florbetapir or ^18^F‐florbetaben PET, respectively. For ADNI, images were processed by the ADNI PET core at UC Berkeley to calculate CLs as previously described.^16^, ^17^ For Penn ADRC, visual reads of positivity/negativity were performed by an experienced nuclear medicine physician/neuroradiologist (I.M.N). Tau PET was collected 75 to 105 minutes after injection of ^18^F‐flortaucipir. For ADNI, processed images with uniform 6 mm full‐width‐at‐half‐maximum resolution were downloaded from the ADNI archive (“Coreg, Av, Std Img and Vox Size, Uniform Resolution”). For Penn ADRC, an in‐house pipeline was used to replicate ADNI processing steps as previously described.^13^ Pre‐processed Tau PET data was then used to calculate global Tau‐MaX for all participants as previously described.^13^ Briefly, Tau‐MaX provides a single global measure of tau burden encompassing both magnitude and extent of tau deposition using a Gaussian mixture‐model approach with values ranging between 0 and 100 with greater values indicating higher tau burden.^13^

### Plasma Collection and Analysis

Samples were collected as previously described for ADNI and Penn ADRC.^18^, ^19^ All samples were analyzed using the Fujirebio Lumipulse G1200 analyzer at either the Penn ADNI core biomarker laboratory (n = 1927 samples) or University of Indiana Biomarker Assay Laboratory (n = 1,154 samples) to measure concentrations of p‐tau_217_, Aβ_42_, and Aβ_40_.

### Plasma Cutpoint Determination

Prior to generating biomarker trajectories, we generated single cutpoints for amyloid PET (Centiloids >24.4) and tau PET (Tau‐MaX >3.31) positivity for plasma biomarkers using data from individuals with both modalities available. Prior to cutpoint determination, we held out 20% of data and performed biomarker development on the remaining 80% using 5‐fold partitioning into training‐validation sets. Receiver operating curve (ROC) analysis was performed on each training fold and the cutpoint with the maximum Youden Index was selected and then applied to corresponding validation dataset. We then selected the cutpoint with the maximal accuracy in its validation dataset. Finally, the selected cutpoints were applied to the held out 20% of data to assess performance on fully unseen data. For amyloid status, cutpoints were generated for both p‐tau_217_/Aβ_42_ (positivity >0.00576) and p‐tau_217_ (positivity >0.1595). For tau status, a cutpoint was generated for p‐tau_217_ (positivity >0.317).

### SILA

We generated separate biomarker trajectories for PET and plasma biomarkers of amyloid and tau using the *silaR* implementation of SILA in R version 4.5.1.^10^ For the amyloid PET model, all measurements from participants with at least 2 scans were included and the threshold for positivity was set to CL >24.4 based on prior post‐mortem validation.^20^ For the tau PET model, all measurements from Aβ+ participants (CL >24.4 or positive visual read) with at least 2 Tau‐MaX measurements were included, and the threshold for positivity was set to Tau‐MaX >3.31, which represents the 97.5^th^ percentile of cognitively unimpaired (CU) Aβ negative (Aβ−) participants from our prior dataset.^13^ For the amyloid plasma models, two separate models (p‐tau_217_/Aβ_42_ and p‐tau_217_) were generated using all participants with at least 2 plasma measurements available and Aβ positive thresholds described above. For the tau plasma model, all Aβ+ participants (p‐tau_217_ >0.1595) with at least 2 p‐tau_217_ measures were included, and the tau positive threshold described above was used.

Prior to running SILA models, data were windsorized at CL >200 = 200, Tau‐MaX >90 = 90, and p‐tau_217_ >2.5 = 2.5, which represented approximate values at which measures were equally likely to go back down rather than continue to rise based on visual inspection and measurement of variance in the rate of change. SILA models were then run using an integration step‐size of 1 year with 100 maximum iterations. We then used these trajectories to estimate amyloid and tau onset age for all participants with at least one measure for a given modality using values from the last available time point with up to 3 years of extrapolation if values were outside of the modeled trajectories. Due to the limited dynamic range in the p‐tau_217_/Aβ_42_ ratio, we ultimately opted to use the p‐tau_217_ plasma amyloid model in our primary analyses, but p‐tau_217_/Aβ_42_ model and performance are show in the Supplemental Material. Thus, four estimated onset ages were calculated: (1) PET‐estimated Aβ onset, (2) plasma‐estimated Aβ onset, (3) PET‐estimated tau onset, and (4) plasma‐estimated tau onset.

To ensure the robustness of SILA trajectory and age estimates, we performed 5‐fold validation with generation of the SILA models in the training set then used to estimate age in the corresponding testing dataset. We used Pearson correlation between estimated onset ages across the folds to evaluate the robustness of estimates across different training and application sets (see Supplemental Material for SILA trajectories).

### Statistical Analysis

R version 4.5.1 was used for all statistical analyses. False discovery rate (FDR) was used for multiple comparison correction with corrected *p* < 0.05 considered significant.

#### Calculating Actual Onset Ages

Actual amyloid onset age (AOA) and tau onset age (TOA) were calculated in a subset of participants who converted from biomarker negative to biomarker positive during longitudinal follow‐up. Actual onset age was calculated using an interval‐censored model using the last known biomarker negative date and first known biomarker positive date as inputs to impute the actual onset age using the accelerated failure time model in the *icenReg* package.^21^ Sensitivity analyses used multiple interval‐censoring approaches using left, right, midpoint, and random selection to determine actual onset age (Table S1).

#### Comparing Plasma and PET‐Based Models Overlap and Accuracy

Pearson correlation analyses evaluated the association between PET‐ and plasma‐estimated amyloid or tau onset ages, respectively, across all participants and within Aβ+ individuals. We then evaluated this association exclusively within participants who were not included in biomarker cutpoint. Next, Spearman correlation and linear regression evaluated the association between estimated and actual onset ages. Mean absolute error (MAE) was calculated for each regression to assess prediction accuracy. Mean and absolute difference between SILA‐estimated onset ages and actual onset ages were also calculated for all measures and comparisons were performed using independent samples t‐tests with unequal variance.

#### Examining Factors Influencing Estimated Tau Onset and Tau Onset to Dementia Gap

We next examined factors influencing estimated tau onset in Aβ+ individuals using Cox‐proportional hazards models for plasma and PET models separately. We tested both univariable and multivariable models to evaluate the influence of estimated amyloid onset, sex, ApoE status, and education on estimated tau onset. ApoE status was divided into two groups based on the presence or absence of ≥1 ε4 allele, and education was divided into three groups: high school (HS) with ≤12 years, college >12 but ≤16, and graduate (Grad) with >16. Finally, we investigated factors influencing time from estimated tau onset to dementia onset in amyloid+ individuals. Time from tau onset was calculated for every visit and time of dementia was considered to be the first time of dementia diagnosis, right censored at time of last follow‐up visit, or left‐censored in those who had a diagnosis of dementia at baseline. Both univariable and multivariable Cox‐proportional hazards models were used for plasma and PET models separately to test the influence of estimated tau onset, sex, ApoE status, and education on tau onset to Dementia time.

## Results

Overall, there were 1733 individuals with CL available, 1,087 with Tau‐MaX available (ADNI = 875, Penn ADRC = 212), and 1,655 individuals with plasma p‐tau_217_ (ADNI = 1252, Penn ADRC = 403). There were a total of 1,097 individuals with longitudinal CL available with mean 3.05 ± 1.26 amyloid PET scans over 4.77 ± 2.95 years. There were 230 Aβ+ individuals with longitudinal Tau‐MaX available with mean 2.85 ± 0.92 tau PET scans over 3.32 ± 1.94 years. For plasma, there were 752 total individuals with longitudinal data available with mean 2.74 ± 0.71 samples over 5.06 ± 2.77 years, while 413 Aβ+ (p‐tau_217_ >1,595) individuals had longitudinal data with mean 2.56 ± 0.72 samples over 4.33 ± 2.69 years. The demographics for the overall and longitudinal (developmental) samples are shown in Table 1.

**TABLE 1:** Cohort Characteristics

Parameter	SILA Development Dataset				Full Dataset		
	Amyloid PET n = 1,097	Tau PET n = 230	Plasma Amyloid n = 752	Plasma Tau n = 413	Amyloid PET n = 1733	Tau PET n = 1,087	Plasma Cohort n = 1,655
Age	77.3 (7.71)	77.6 (7.21)	77.8 (7.31)	79.4 (7.43)	75.8 (8.08)	74.8 (7.79)	75.4 (7.87)
Sex							
F	533 (48.6%)	120 (52.2%)	366 (48.7%)	187 (45.3%)	851 (49.1%)	599 (55.1%)	857 (51.8%)
M	564 (51.4%)	110 (47.8%)	386 (51.3%)	226 (54.7%)	882 (50.9%)	488 (44.9%)	798 (48.2%)
Education (years)	16.4 (2.57)	16.2 (2.38)	16.3 (2.62)	16.2 (2.66)	16.3 (2.57)	16.4 (2.45)	16.3 (2.55)
Amyloid							
Positive	546 (49.8%)	230 (100%)	413 (54.9%)	413 (100%)	881 (50.8%)	464 (44.7%)	858 (51.8%)
Negative	551 (50.2%)	0	339 (45.1%)	0	853 (49.2%)	573 (55.3%)	797 (48.2%)
ApoE ε4							
Absent	615 (58.9%)	80 (38.8%)	449 (62.3%)	198 (49.5%)	891 (57%)	577 (61.9%)	864 (57.8%)
Present	429 (41.1%)	126 (61.2%)	272 (32.0%)	202 (50.5%)	672 (43%)	355 (38.1%)	632 (42.2%)
Race							
Black	58 (5.3%)	18 (7.8%)	75 (10.0%)	27 (6.5%)	127 (7.3%)	185 (17.3%)	239 (14.7%)
White	993 (90.5%)	203 (88.3%)	652 (86.7%)	377 (91.3%)	1,521 (87.8%)	850 (79.3%)	1,349 (82.8%)
Other*	26 (4.2%)	9 (3.9%)	25 (3.3%)	9 (2.2%)	85 (4.9%)	37 (3.4%)	42 (2.6%)
Diagnosis							
CU	499 (45.5%)	104 (45.2%)	382 (50.8%)	140 (33.9%)	689 (40.0%)	635 (58.8%)	848 (51.5%)
MCI	380 (34.6%)	58 (25.20%)	216 (28.7%)	132 (32.0%)	635 (36.8%)	295 (27.3%)	470 (28.5%)
Dementia	218 (19.9%)	68 (29.6%)	154 (20.5%)	141 (34.1%)	400 (23.2%)	150 (13.9%)	330 (20.0%)

Mean (SD) for continuous measures and count (%) for categorical variables. Age is baseline age for SILA development dataset and age at last time point (used to estimate onset) in full dataset.

ApoE = apolipoprotein E; CU = cognitively unimpaired; F = Female; M = Male; MCI = mild cognitive impairment; SILA = sampled‐iterative local approximation.

### Plasma Cutpoint Determination

There were 1,138 individuals with both CL and plasma p‐tau_217_ and p‐tau_217_/Aβ_42_ ratio available. For p‐tau_217_, accuracy in the training folds ranged from 82.6 to 84.3% and from 81.2 to 88.5% in the corresponding test folds with the optimal cutpoint determined to be p‐tau_217_ >0.1595. For p‐tau_217_/Aβ_42_ accuracy in the training folds ranged from 84.8 to 86.7% and from 84.4 to 90.3% in the corresponding test folds with the optimal cutpoint determined to be p‐tau_217_/Aβ_42_ >0.00576. There were 1,012 individuals with both Tau‐MaX and plasma p‐tau_217_ available. Accuracy in the training folds ranged from 84.2 to 87.7% in the training folds and 81.8 to 91.7% in the testing folds with the optimal cutpoint determined to be p‐tau_217_ >0.317. Performance of each measure is shown in Table 2.

**TABLE 2:** Plasma Cutpoints and Performance in Held‐Out Data

Parameter	Value	Accuracy	Sensitivity	Specificity	PPV	NPV
Amyloid PET						
p‐tau_217_	0.1595 pg/μL	87.1%	93.4%	79.4%	84.5%	90.9%
p‐tau_217_/Aβ_42_	0.00576	89.9%	97.3%	81.5%	85.7%	96.4%
Tau PET						
p‐tau_217_	0.317 pg/μL	86.8%	93.8%	67.4%	88.9%	79.5%

Aβ = amyloid‐β; NPV = negative predictive value; PET = positron emission tomography; PPV = positive predictive value.

### SILA Models of Amyloid Trajectory

**FIGURE 1:**
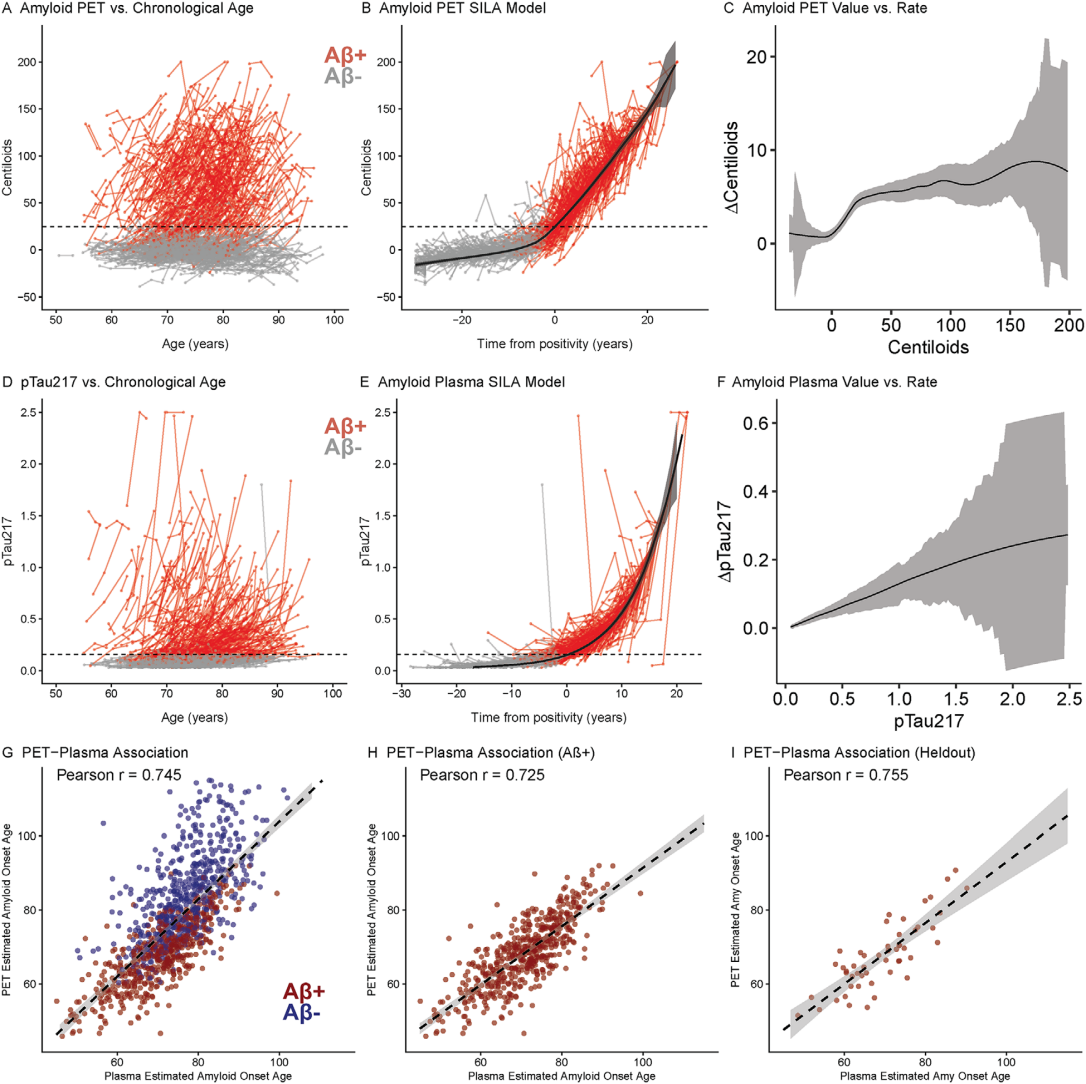
Temporal modeling of amyloid trajectories using PET and plasma. Longitudinal biomarker trajectories against chronological age (A, D), time from biomarker positivity (B, E) with each point representing a single timepoint, lines connecting points from the same participant, and color/shading delineating Aβ+ and Aβ‐ participants. Dashed horizontal lines indicate amyloid positivity threshold. B, E: SILA trajectory curves are shown with shading representing the error. C, F: Estimated rate of change for a given CL (C) or p‐tau_217_ (F) value is shown with error shown by the shaded ribbon. G–I: Association between PET‐ and plasma‐estimated amyloid onset in all individuals (G), within Aβ+ individuals (H), and Aβ+ individuals that were heldout from cutpoint development (I). The dashed line represents the linear best‐fit with 95% confidence of fit shown by the shaded ribbon. Color/shading delineates Aβ+ participants and Aβ‐ participants. Aβ = amyloid‐β; PET = positron emission tomography; SILA = sampled‐iterative local approximation. [Color figure can be viewed at www.annalsofneurology.org]

**FIGURE 2:**
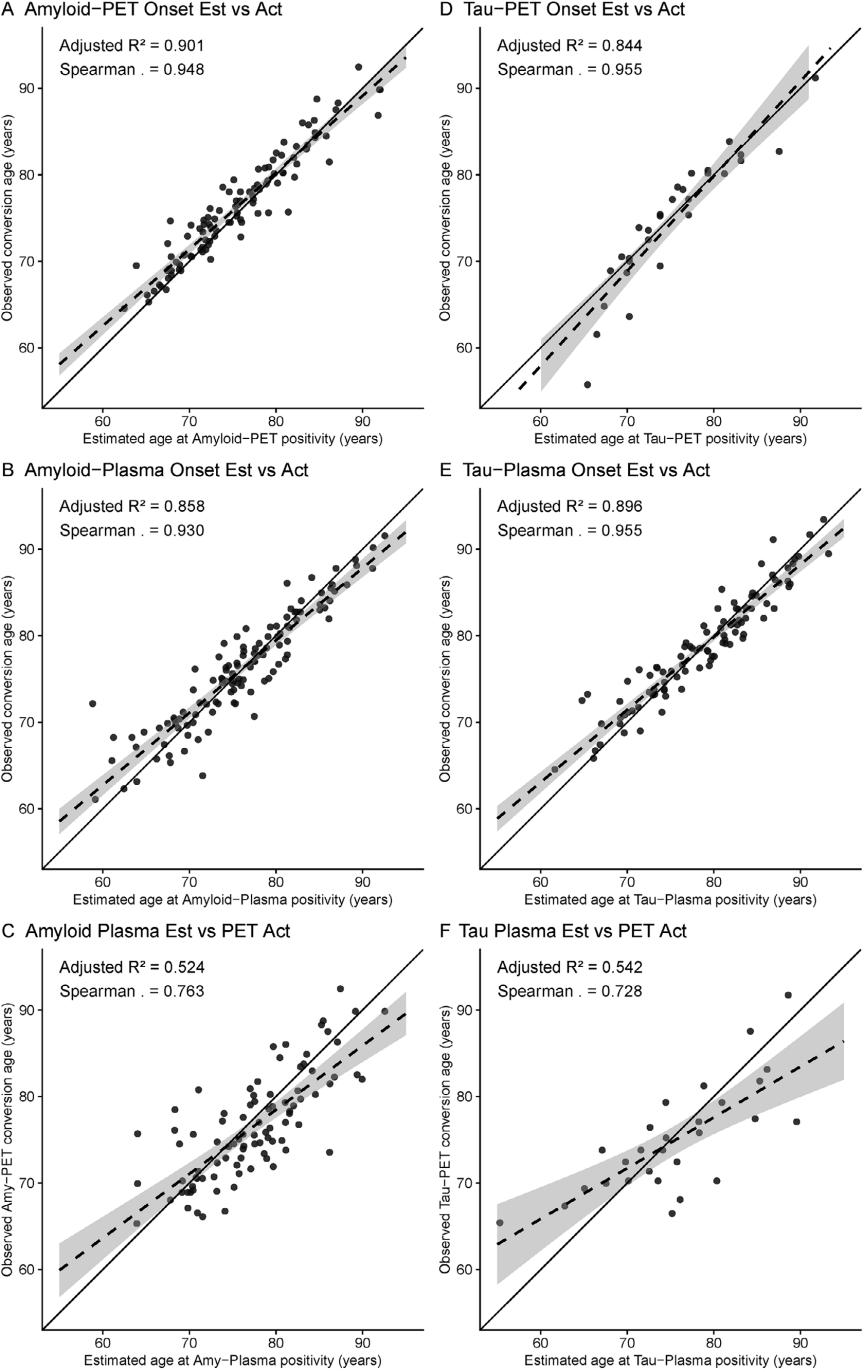
Comparison of estimated and actual onset age. Associations of estimated and actual onset for PET (A, D), plasma (B, E), and estimated plasma to actual PET conversion (C, F) in a subset of individuals who converted from Amyloid (A–C) or Tau (D–F) negative to positive during follow‐up. The dashed line represents the linear best‐fit with the ribbon showing the 95% confidence of fit, while the solid line is the identity line shown for reference (slope = 1). The R^2^ and Spearman correlation coefficient are shown. PET = positron emission tomography.

The amyloid PET SILA trajectory and ΔCL by CL curves are shown in Figure 1A–C. Average PET‐estimated amyloid onset was 76.2 ± 14.7 years for all individuals and 66.8 ± 9.50 years for Aβ+ individuals. The plasma amyloid SILA trajectory and Δp‐tau_217_ by p‐tau_217_ curves are shown in Figure 1D–F. Average plasma‐estimated amyloid onset was 73.6 ± 9.66 years for all individuals and 68.5 ± 9.44 years for Aβ+ individuals. Cross‐validation models (Figure S1) revealed high correlation of estimated amyloid onset age between different training‐test folds when using either PET (mean *r* = 0.995, range: 0.989–0.999) or plasma (mean *r* = 0.999, range: 0.997–0.999) models. In the 1,138 individuals with both CL and p‐tau_217_ available, there was a strong correlation between PET and plasma‐estimated onset ages (*r* = 0.75, *df* = 1,136, *p* < 0.001) in all individuals, in the 615 Aβ+ individuals (*r* = 0.72, *df* = 613, *p* < 0.001), and in the 60 Aβ+ individuals not included in cutpoint determination (*r* = 0.76, *df* = 58, *p* < 0.001) (Fig 1G–I). There were 103 individuals in the Amyloid PET dataset, 110 individuals in the plasma dataset, and 94 in the combined dataset that converted from Aβ− to Aβ+ during longitudinal follow‐up. There was strong association between estimated and actual amyloid onset (Fig 2A–C) for PET (*β* = 0.95 [0.89, 1], *p* < 0.001, MAE = 1.50), plasma (*β* = 0.93 [0.86, 1], *p* < 0.001, MAE =1.85), and for estimated plasma to actual PET onset (*β* = 0.73 [0.59, 0.87], *p* < 0.001, MAE = 3.42). The mean difference between estimated and actual onset was significantly higher for PET compared to plasma models (*t* = 2.10, *df* = 210.5, *p* = 0.037, Table 3), while the absolute mean difference was slightly larger for plasma compared to PET (*t* = −1.71, *df* = 212.9, *p* = 0.088).

**TABLE 3:** SILA Models Prediction Error for Actual Onset Age

Parameter	PET‐Estimated Amyloid Onset	Plasma‐Estimated Amyloid Onset	PET‐Estimated Tau Onset	Plasma‐Estimated Tau Onset	Plasma‐PET Comparison	
					Amyloid	Tau
MAE	1.50	1.84	2.03	1.56	‐‐	‐‐
Cross‐modal MAE	‐‐	3.42	‐‐	3.09	‐‐	‐‐
mean difference	0.75	0.06	0.61	0.03	*t* (210.5) = 2.10, *p* = 0.037	*t* (38.4) = 0.86 *p* = 0.34
Mean absolute difference	1.63	2.02	2.16	1.74	(*t* (212.9) = −1.71, *p* = 0.088)	*t* (36.5) = 0.99 *p* = 0.33

*Note:* T‐tests comparing error of PET and plasma models for amyloid and tau.

MAE = mean absolute error; PET = positron emission tomography; SILA = sampled‐iterative local approximation.

### SILA Models of Tau Trajectory

**FIGURE 3:**
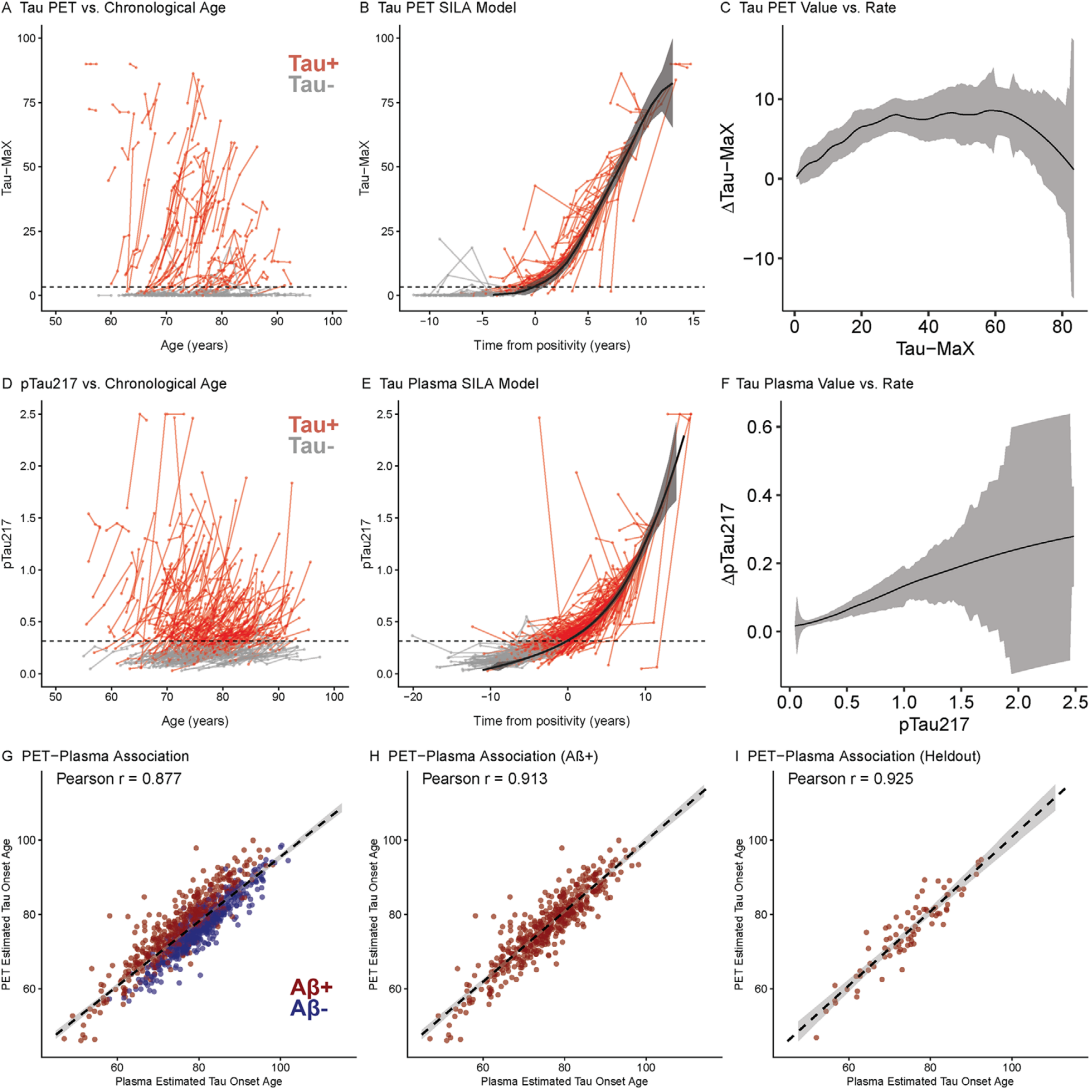
Temporal modeling of tau trajectories using PET and plasma. Longitudinal biomarker trajectories against chronological age (A, D), time from biomarker positivity (B, E) with each point representing a single timepoint, lines connecting points from the same participant, and color/shading delineating Tau+ and Tau‐ participants. Dashed horizontal lines indicate tau positivity threshold. B, E: SILA trajectory curves are shown with shading representing the error. C, F: Estimated rate of change for a given Tau‐MaX (C) or p‐tau_217_ (F) value is shown with error shown by the shaded ribbon. G‐I: Association between PET‐ and plasma‐estimated tau onset in all individuals (G), within Aβ+ individuals (H), and Aβ+ individuals that were heldout from cutpoint development (I). The dashed line represents the linear best‐fit with 95% confidence of fit shown by the shaded ribbon. Color/shading delineates Aβ+ and Aβ‐ participants. Aβ = amyloid‐β; PET = positron emission tomography; SILA = sampled‐iterative local approximation. [Color figure can be viewed at www.annalsofneurology.org]

The tau PET SILA trajectory and ΔTau‐MaX by Tau‐MaX curves are shown in Figure 3A–C. Average PET‐estimated tau onset was 76.5 ± 8.54 years for all individuals, 75.9 ± 9.56 years for Aβ+ individuals, and 71.4 ± 9.78 years for Tau+ (Tau‐MaX >3.31) individuals. The plasma tau SILA trajectory and Δp‐tau_217_ by p‐tau_217_ curves are shown in Figure 3D–F. Average plasma‐estimated tau onset was 78.0 ± 8.96 years across all individuals, 74.3 ± 9.36 for Aβ+ individuals, and 71.0 ± 9.31 for Tau+ (p‐tau_217_ >0.317) individuals. Cross‐validation models (Figure S1) revealed high correlation of estimated tau onset age between different training‐test folds when using either PET (mean *r* = 0.872, range: 0.814–0.909) or plasma (mean *r* = 0.998, range: 0.996–0.999) models. There was a strong correlation between PET‐ and plasma‐estimated tau onset (Fig 3G–I) in the 1,012 individuals with both Tau‐MaX and p‐tau_217_ available (*r* = 0.88, *df* = 986, *p* < 0.001), within the 378 Aβ+ individuals (*r* = 0.91, *df* = 484, *p* < 0.001) and in the 79 Aβ+ individuals not included in cutpoint determination (*r* = 0.92, *df* = 77, *p* < 0.001). There were 29 individuals in the Tau PET dataset, 84 individuals in the plasma dataset, and 29 in the combined dataset that converted from Tau− to Tau+ during longitudinal follow‐up. Within this group, there was strong association between estimated and actual tau onset (Fig 2D–F) for PET (*β* = 0.92 [0.77, 1], *p* < 0.001, MAE = 2.03), plasma (*β* = 0.95 [0.88, 1], *p* < 0.001, MAE =1.57), and for estimated plasma to actual PET onset (*β* = 0.75 [0.48, 1], *p* < 0.001, MAE = 3.09). Neither the mean or absolute difference between estimated and actual tau onset differed between PET and plasma models (Table 3).

### Factors Influencing Estimated Tau Onset

**FIGURE 4:**
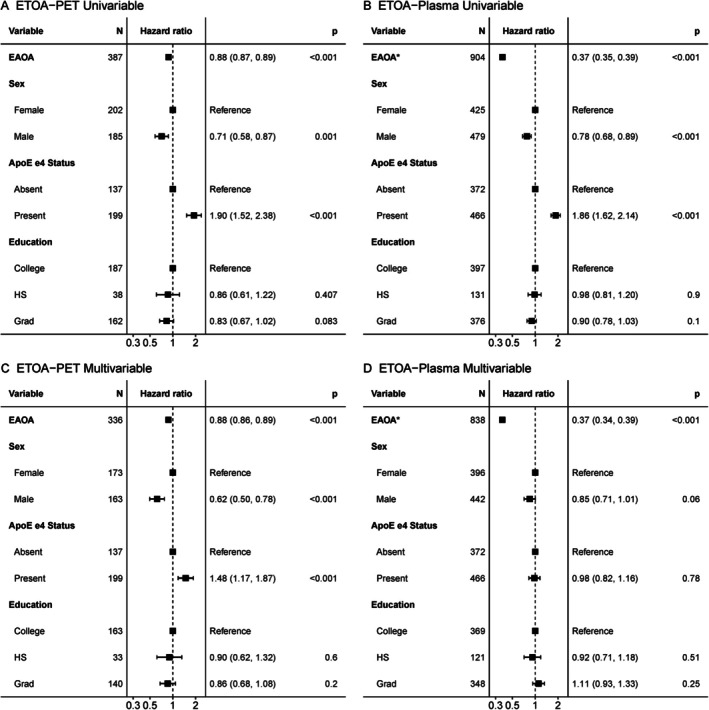
Factors influencing ETOA. Univariable (A) and multivariable (B) cox‐proportional hazards models examining the effects of various factors on ETOA from PET (left) and plasma (right) models. EAOA is from within‐modality. Hazard ratios from Cox‐proportional hazards are shown with 95% confidence intervals and uncorrected p‐values. *p_*FDR*_ <0.05. EAOA = estimated amyloid onset age; ETOA = estimated tau onset age; PET = positron emission tomography.

We first examined univariable models to assess factors influencing PET‐ and plasma‐estimated tau onset separately in Aβ+ individuals (see Supplement for Kaplan–Meier curves). For both the PET (Fig 4A) and plasma (Fig 4B) models, younger estimated tau onset was associated with younger estimated amyloid onset (*c*‐stat = 0.80 [0.77, 0.82] and 0.77 [0.74, 0.79], respectively), female sex (*c*‐stat = 0.56 [0.53, 0.58] and 0.54 [0.52, 0.57], respectively), and presence of at least one ApoE ε4 allele (*c*‐stat = 0.60 [0.57, 0.63] and 0.59 [0.56, 0.62], respectively). We then tested multivariable models with all measures included simultaneously and found that all results from univariable models remained significant in the PET multivariable model (Fig 4C), such that earlier estimated amyloid onset, female sex, and presence of at least one ApoE ε4 allele were all associated with earlier estimated tau onset (*c*‐stat = 0.80 [0.78, 0.82]). For and the multivariable plasma model (Fig 4D), only younger estimated amyloid onset predicted younger estimated tau onset, while female sex trended towards earlier tau onset (*c*‐stat = 0.99 [0.99, 1]).

### Factors Influencing Estimated Tau Onset to Dementia Gap

**FIGURE 5:**
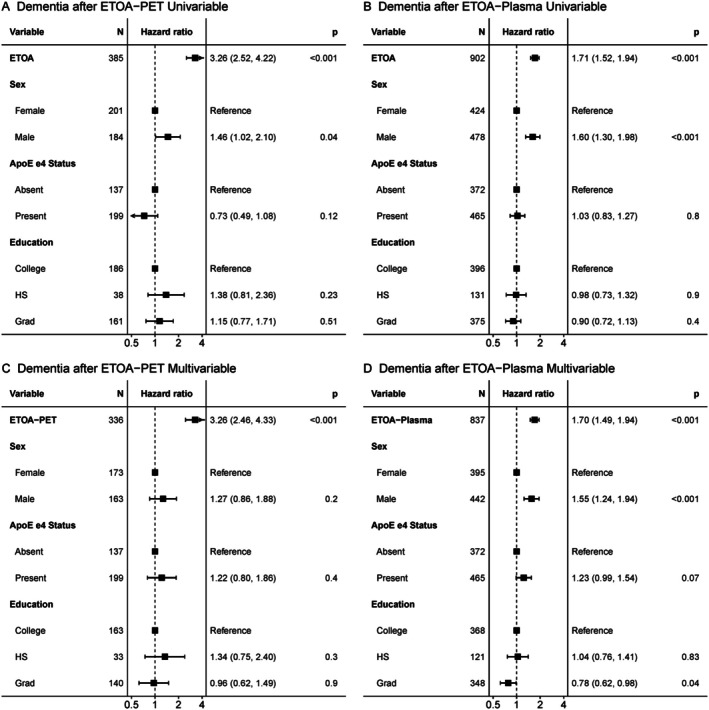
Factors influencing time from ETOA to dementia. Univariable (A) and multivariable (B) Cox‐proportional hazards models examining the effects of various factors on time from ETOA from PET (left) and plasma (right) to dementia diagnosis. To allow for comparability to other factors, ETOA is scaled to represent the risk of every 10 year change. Hazard ratios from Cox‐proportional hazards are shown with 95% confidence intervals and uncorrected p‐values. *p_*FDR*_ <0.05. ETOA = estimated tau onset age.

Next, we evaluated the factors influencing time from estimated tau onset‐to‐dementia onset in Aβ+ individuals. In the Tau‐PET cohort 109/336 Aβ+ individuals had a diagnosis of dementia during follow‐up, while in the p‐tau_217_ cohort 352/837 Aβ+ individuals had a diagnosis of dementia during follow‐up. Univariable models found that younger estimated tau onset (*c*‐stat = 0.78 [0.73, 0.82] and 0.69 [0.66, 0.72], respectively) and female sex (*c*‐stat = 0.59 [0.53, 0.64] and 0.58 [0.55, 0.61], respectively) was associated with longer estimated tau onset to dementia gap in both the PET (Fig 5A) and plasma (Fig 5B) models (see Supplemental Material for Kaplan–Meier curves). For the multivariable PET model (Fig 5C), only earlier estimated tau onset associated with longer estimated tau onset to dementia gap (*c*‐stat = 0.78 [0.73, 0.83]). In contrast, for the multivariable plasma model (Fig 5D) both earlier estimated tau onset and female sex were associated with longer dementia‐free survival after tau‐onset, while there was a trend toward shorter estimated tau onset age (ETOA) to dementia gap in individuals with at least one ApoE ε4 allele and longer ETOA to dementia gap in individuals with graduate education (*c*‐stat = 0.70 [0.67, 0.73]).

## Discussion

Our study extends prior temporal modeling techniques for both amyloid and tau trajectories to plasma‐based models and demonstrates comparability between these two modalities. There was high overlap for both estimated amyloid and tau onset ages between PET and plasma models, although there was slightly higher agreement in tau models. Further, we found largely overlapping factors that influenced estimated tau onset and time from estimated tau onset to dementia between PET‐ and plasma‐based models. Overall, our findings demonstrate that plasma‐based temporal modeling of amyloid and tau trajectories place individuals on the biological timeline of AD with minimal loss compared to PET‐based estimates and, thus, provide a highly accessible alternative approach.

Our amyloid PET model is similar to recently developed SILA models using CL, although we opted for a higher CL cutoff of 24.4 based on prior post‐mortem work and real‐world data that suggest that these higher CL cutoffs are closely aligned with the presence of AD pathology at autopsy and PET visual reads.^20^, ^22^ As a result, the mean PET‐estimated amyloid onset in this study is very similar to that previously identified using the largely overlapping ADNI PET data but with a CL >19 considered abnormal,^12^ as well as similar to prior studies using both SILA and other temporal modeling approaches with standardized uptake value ratio (SUVR) or Pittsburgh Compound B (PiB) distribution volume ratio (DVR).^10^, ^23^, ^24^ However, we extend the SILA approach beyond amyloid PET to a global measure of tau burden using Tau‐MaX, as well as to a plasma biomarker of amyloid and tau, p‐tau_217_. Two recent studies applied temporal modeling to Tau‐PET but used temporal meta–region of interest (ROI) SUVR, which is less sensitive to non‐canonical patterns of tau accumulation and appears to be more susceptible to ceiling effects, particularly with ^18^F‐flortaucipir.^12^, ^13^, ^23^ These findings further highlight the flexibility of temporal modeling to place individuals on the AD pathological timeline using different biomarkers and modalities.

Importantly, there was high overlap between estimated amyloid and tau onset estimated by PET and plasma biomarkers, particularly within Aβ+ individuals. Further, this association was higher for estimated tau onset than estimated amyloid onset, consistent with a stronger association between plasma p‐tau_217_ and Tau‐PET compared to amyloid PET, despite p‐tau_217_ frequently being thought of as a marker specifically of amyloid positivity.^13^, ^18^, ^25^ When examining average onset age across all measures, we found that biomarkers became abnormal in the following order: (1) amyloid PET, (2) p‐tau_217_ Aβ cutoff, (3) p‐tau_217_ tau cutoff, (4) Tau PET, which is consistent with the current understanding of the AD pathological cascade with accumulation of amyloid plaques leading to abnormal phosphorylation of soluble tau species and then formation of insoluble tau neurofibrillary tangles that can be measured with Tau PET.^3^ The degree to which each marker represents slightly different events in the neuropathological cascade may also be the primary reason for considerably greater error when attempting to predict onset across modalities despite high correlation. This is consistent with a similar approach examining multiple plasma p‐tau species with PET‐based stages of amyloid and tau pathology.^23^

In addition to high overlap between PET and plasma estimated amyloid and tau onset ages, we also found that using PET or plasma‐based estimates resulted in similar factors influencing estimated tau onset. Specifically, we found that earlier estimated amyloid onset, female sex, and presence of at least one ApoE ε4 allele were associated with earlier estimated tau onset in both univariable and multivariable models. These findings are consistent with studies showing that early‐onset AD is associated with earlier (and greater) tau accumulation compared to late‐onset AD^26^, women have higher tau loads for a given level of amyloid compared to men,^26^, ^27^ and ApoE plays an important role in tau accumulation that is independent of amyloid.^28^, ^29^ Further, these findings are similar to a prior study using SILA to investigate factors influencing estimated tau onset age.^12^

In contrast to factors influencing estimated tau onset age, multivariable models investigating the factors associated with dementia‐free survival following estimated tau onset showed larger differences between PET and plasma models. While PET models showed that only earlier estimated tau onset age was associated with longer dementia free survival after tau onset, plasma models found that both estimated tau onset and sex had significant effects, while education and ApoE status had marginal effects. One previous study has found similar associations between earlier estimated tau onset age and dementia‐free survival after tau onset using PET.^12^ However, our plasma findings are consistent with prior work showing that individuals with early‐onset AD tend to have onset of symptoms at much later biological disease stage compared to late‐onset cases,^26^, ^30^ women have later biological disease stage at symptom onset compared to men,^27^, ^31^, ^32^ and those with at least one ApoE ε4 allele have faster cognitive decline than non‐carriers.^31^, ^33^, ^34^ It should be noted that the age and sex differences could be related to differences in co‐pathology, which likely plays an important role in the gap between tau onset and all‐cause dementia with shorter gaps in those with co‐pathology.^35^, ^36^ Similarly, a large body of research in cognitive reserve has demonstrated that factors such as education are associated with higher resilience to tau pathology with later onset of cognitive symptoms.^37^ Of note, these findings in the plasma model are likely related to stronger power, as all effects except for estimated tau onset disappear when restricted to an overlap cohort with both tau PET and plasma.

This study has several limitations. First, due to lack of alternative plasma biomarkers and poorer dynamic range for the p‐tau_217_/Aβ_42_ ratio, we used the same measure to estimate estimated amyloid and tau onset in blood, thus creating a stronger dependence of tau onset on amyloid onset. We did alter the samples used to generate these trajectories, but there is still significant overlap between these models. From a biological perspective, p‐tau_217_ clearly captures aspects of both amyloid and tau pathology, making it sensitive but less specific for either of these pathologies individually. Development of more specific biomarkers of tau, such as microtubule‐binding region containing tau residue 243 (MBTR‐tau243), may help in isolating tau‐specific and amyloid‐specific aspects of p‐tau_217_ to further refine these models.^38^ Second, our assessment of factors influencing estimated tau onset and time from estimated tau onset to dementia was relatively limited to measures widely available within these datasets. Future work will be necessary to more fully evaluate the influence of various factors, particularly those associated with genetics and social determinants of health, as well as further examination of factors that may contribute to individual error in estimated amyloid onset age (EAOA) and ETOA prediction. Finally, plasma biomarkers are susceptible to systemic disease and comorbidity, particularly renal dysfunction, which these studies do not adequately capture.^39^, ^40^ Given the complications of accurately assessing plasma biomarkers in individuals with significant kidney disease, these individuals may be better suited to PET‐based estimates. However, ongoing work continues to investigate approaches for correcting for renal dysfunction and future work should compare trajectories using corrected and uncorrected values.^41^

A major strength of this study is the application of temporal modeling to 2 new domains: (1) global tau burden measured by Tau‐PET using Tau‐MaX and (2) plasma p‐tau_217_. While temporal modeling has been more thoroughly examined in the context of amyloid, including recent attempts to use plasma ratios of p‐tau_217_ for modeling amyloid, these models of tau burden represent an important advance given tau's close association with cognition and major role in biological staging. These models provide valuable tools for anchoring the disease course to multiple biological milestones without needing to directly observe these transitions during longitudinal follow‐up and move beyond simple dichotomous designations of positive and negative or discrete tau stages. Further, the estimation of an age of onset rather than raw biomarker measurement values (eg, SUVR) provides a more conceptually tractable tool for understanding biological stage. This may be of particular value when assessing individuals clinically, who often do not present until after the onset of cognitive symptoms. In this setting, obtaining p‐tau_217_ could allow for placing an individual on the amyloid and tau timeline, and, after combining this with their demographics and ApoE status, provide prognostic guidance for likely time to dementia onset. This may be of even greater importance in the era of disease‐modifying therapies, where providing a general sense of an individual's time from estimated tau onset and development of dementia could be valuable when making treatment decisions. Furthermore, large discrepancies between clinical severity and what would be expected at a given biological disease duration may suggest presence of co‐pathology or resilience,^8^, ^9^, ^42^ which again may impact treatment decisions.

In conclusion, we generated temporal models of amyloid and tau trajectories for both PET and plasma markers of pathology. These models show high overlap and comparable error, particularly for predicting tau onset. Furthermore, this approach helps to further elucidate the important role of age of amyloid onset, sex, and ApoE status in disease trajectory. Together, these findings highlight the potential value of temporal modeling approaches and their direct ability for translation to clinic via widely collected plasma biomarkers of AD pathology.

## Author Contributions

Conception and design of the study: C.A.B., K.A.Q.C., A.C.P., J.A.D., C.T.M., E.B.L., D.M.H., P.A.Y., I.M.N., L.M.S., D.A.W. Acquisition and analysis of data: C.A.B., K.A.Q.C., M.K., E.M., S.R.D., D.M.H., I.M.N., L.M.S.

## Potential Conflicts of Interest

C.A.B., K.A.Q.C, M.K., E.M., J.A.D., C.T.M., S.R.D., D.M.H., and P.A.Y. declare no competing interests. Alice Chen‐Plotkin has a patent licensed to Prevail Therapeutics for genetic approaches to treating frontotemporal dementia. Edward Lee has served as a paid consultant for Wavebreak Therapeutics and Eli Lilly. Ilya Nasrallah has served on the scientific advisory board for Eisai and done educational speaking for Biogen. Leslie Shaw has served on scientific advisory boards and/or as a consultant for Biogen, Roche Diagnostics, Fujirebio, Siemens, and Diadem and has given lectures for Biogen, Roche, and Fujirebio. David Wolk has served as a paid consultant for Eli Lilly and Beckman Coulter. He has also served on the DSMB for Functional Neuromodulation and GSK. He has received research support paid to his institution by Biogen.

## Supporting information


**Figure S1.** K‐Fold SILA Trajectories. Longitudinal biomarker trajectories against time from positivity for Amyloid (top) and Tau (bottom) PET (left) and plasma (right) biomarkers across repeated folds. Each fold is shown by a different colored line. The dashed line represents the threshold of biomarker positivity.
**Figure S2.** Plasma p‐tau_217_/Aβ_42_ ratio SILA trajectory. Longitudinal biomarker trajectories against chronological age (A) and time from biomarker positivity (B) with each point representing a single timepoint, lines connecting points from the same participants, and Aβ+ participants shown in red with Aβ‐ shown in gray. Dashed lines indicate the amyloid positivity threshold. B: The SILA trajectory curve is shown. C: Estimated rate of change for a given p‐tau_217_/Aβ_42_ value is shown with error shown by the shaded ribbon.
**Figure S3.** Associations with Plasma p‐tau_217_/Aβ_42_ estimated amyloid onset. A, B: The association between plasma‐estimated and PET‐estimated amyloid onset age is shown in all (A) and Aβ+ (B) participants with Aβ+ individuals shown in red and Aβ‐ shown in blue. The dashed line represents the linear best‐fit with the shaded ribbon indicating the 95% confidence interval for the linear fit. C, D: Association between estimated and actual onset age for within modality (C) and across modality with plasma estimated age predicting actual PET onset (D). The linear best fit is shown by the dashed line with the shaded ribbon showing the 95% confidence of fit, while the solid line represents the identity line for reference (slope = 1). The *R* ^2^ and Spearman correlation are shown.
**Figure S4.** Kaplan–Meier plots of univariable factors influencing estimated tau onset age. Kaplan–Meier curves for the univariable cox‐proportional hazard models for PET (A‐D) and plasma (E‐H) estimated tau onset age are shown. Lines indicate the survival curve and shaded ribbons reflect the 95% confidence interval. The dashed vertical lines indicate the age at which 50% of the individuals have had tau onset. Statistics for these models are reported in the main text in Figure 4. A, E: For models testing the impact of estimated amyloid onset age (EAOA), individuals were grouped for visualization.
**Figure S5.** Kaplan–Meier plots of univariable factors influencing time from tau onset to development of dementia. Kaplan–Meier curves for the univariable cox‐proportional hazard models for time from PET (A–D) and plasma (E–H) tau onset to dementia are shown. Lines indicate the survival curve and shaded ribbons reflect the 95% confidence interval. Censored data is depicted by vertical hashes on the survival curves. The dashed vertical lines indicate the age at which 50% of the individuals have a diagnosis of dementia. Statistics for these models are reported in the main text in Figure 5. A, E: For models testing the impact of estimated tau onset age (ETOA), individuals were grouped for visualization.
**Table S1.** Correlation of estimated onset age with actual onset using different imputation methods.

## Data Availability

All requests for raw and analyzed data from the Penn ADRC cohort will be reviewed by the Penn Neurodegenerative Data Sharing Committee (PNDSC) and shared for appropriate uses through a data sharing agreement (https://www.pennbindlab.com/data-sharing). Anonymized data from Penn ADRC will be shared upon request to the corresponding author by a qualified academic investigator for the purpose of replicating procedures and results in this article. Data are not publicly available due to privacy protections outlined in the participant informed consent. Documents related to study protocols, informed consent and other documentation can similarly be made available upon request. All ADNI data are shared without embargo through the LONI Image and Data Archive (https://ida.loni.usc.edu/), a secure research data repository. Interested scientists may obtain access to ADNI imaging, clinical, genomic, and biomarker data for the purposes of scientific investigation, teaching, or planning clinical research studies. Access is contingent on adherence to the ADNI Data Use Agreement and the publications' policies (https://adni.loni.usc.edu/data-samples/access-data/).
