# Supplementary material for: Temporal Modeling of Amyloid and Tau Trajectories in Alzheimer's Disease Using PET and Plasma Biomarkers
Source: Ann Neurol. 2026 Mar 15;99(6):1438–51. doi: 10.1002/ana.78194 (PMC13206525; doi:10.1002/ana.78194)
Supplement: Supplementary file 1 — Figure S1. K‐Fold SILA Trajectories. Longitudinal biomarker trajectories against time from positivity for Amyloid (top) and Tau (bottom) PET (left) and plasma (right) biomarkers across repeated folds. Each fold is shown by a different colored line. The dashed line represents the threshold of biomarker positivity. Figure S2. Plasma p‐tau217/Aβ42 ratio SILA trajectory. Longitudinal biomarker trajectories against chronological age (A) and time from biomarker positivity (B) with each point representing a single timepoint, lines connecting points from the same participants, and Aβ+ participants shown in red with Aβ‐ shown in gray. Dashed lines indicate the amyloid positivity threshold. B: The SILA trajectory curve is shown. C: Estimated rate of change for a given p‐tau217/Aβ42 value is shown with error shown by the shaded ribbon. Figure S3. Associations with Plasma p‐tau217/Aβ42 estimated amyloid onset. A, B: The association between plasma‐estimated and PET‐estimated amyloid onset age is shown in all (A) and Aβ+ (B) participants with Aβ+ individuals shown in red and Aβ‐ shown in blue. The dashed line represents the linear best‐fit with the shaded ribbon indicating the 95% confidence interval for the linear fit. C, D: Association between estimated and actual onset age for within modality (C) and across modality with plasma estimated age predicting actual PET onset (D). The linear best fit is shown by the dashed line with the shaded ribbon showing the 95% confidence of fit, while the solid line represents the identity line for reference (slope = 1). The R 2 and Spearman correlation are shown. Figure S4. Kaplan–Meier plots of univariable factors influencing estimated tau onset age. Kaplan–Meier curves for the univariable cox‐proportional hazard models for PET (A‐D) and plasma (E‐H) estimated tau onset age are shown. Lines indicate the survival curve and shaded ribbons reflect the 95% confidence interval. The dashed vertical lines indicate the age at which 50% of the individ [file ANA-99-1438-s001.docx]

**
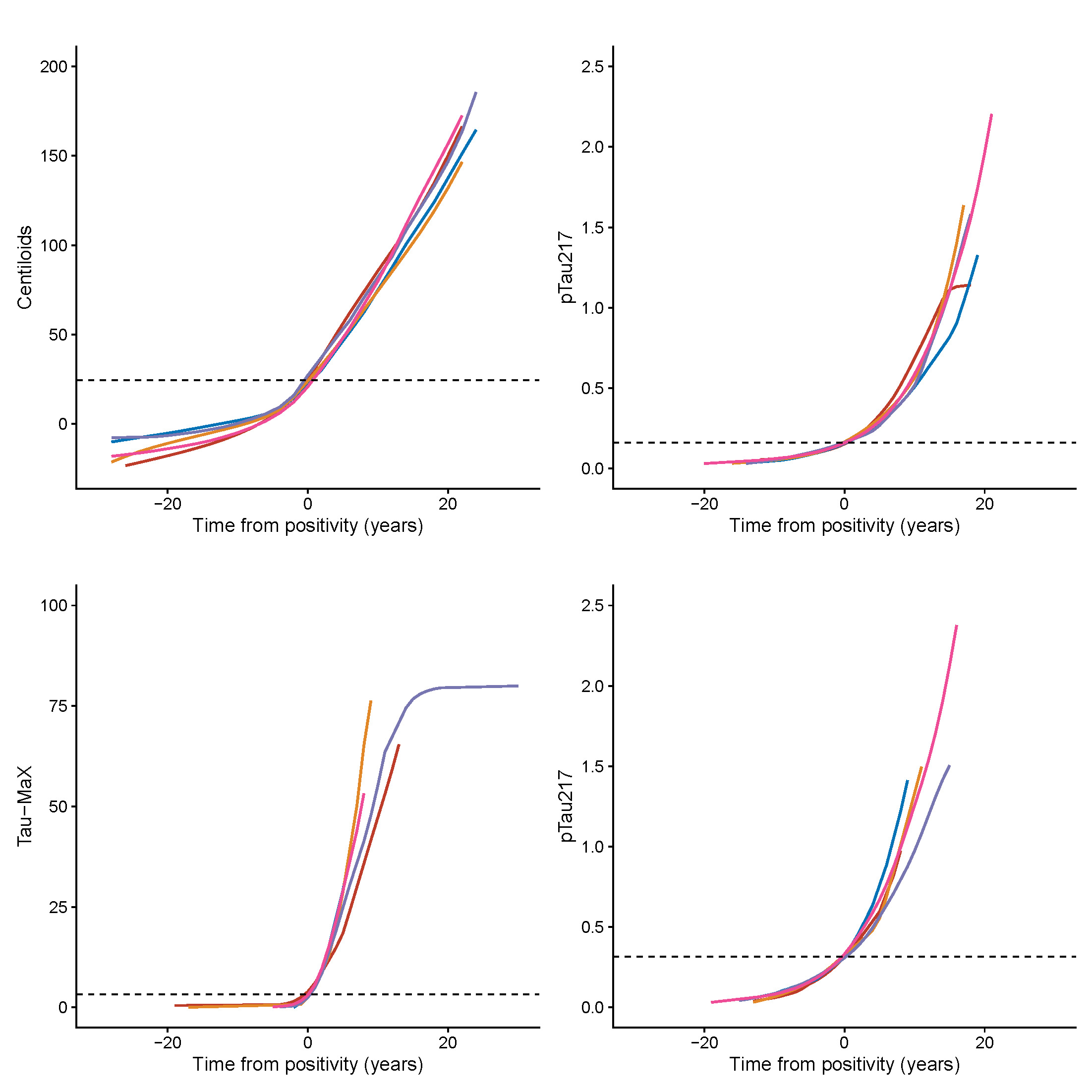
**

**Figure S1. K-Fold SILA Trajectories.** Longitudinal biomarker trajectories against time from positivity for Amyloid (top) and Tau (bottom) PET (left) and plasma (right) biomarkers across repeated folds. Each fold is shown by a different colored line. The dashed line represents the threshold of biomarker positivity.


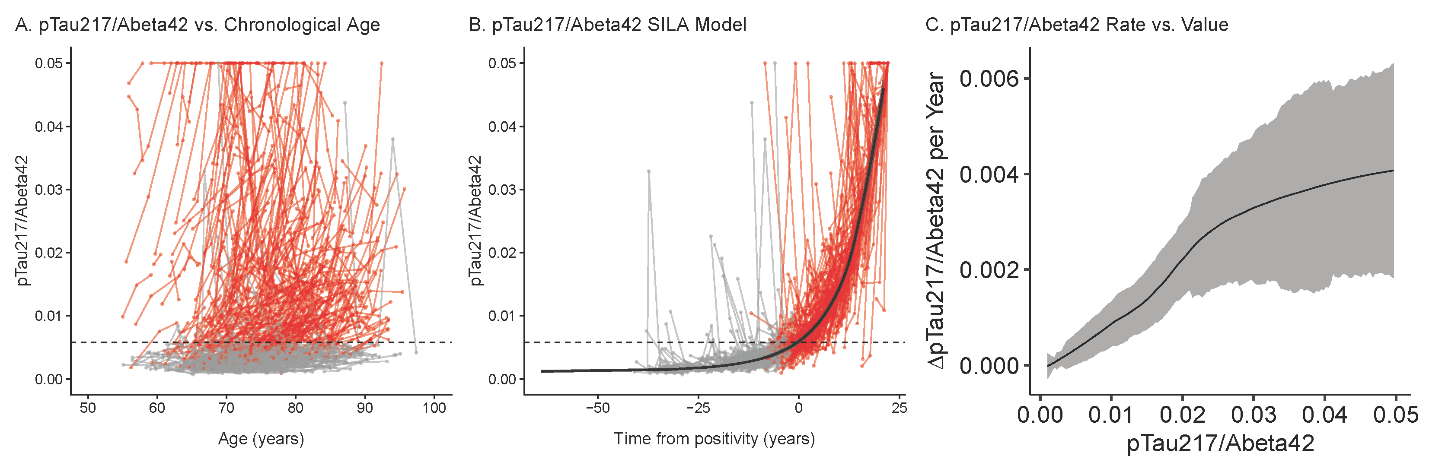


**Figure S2. Plasma p-tau_217_/Aβ_42_ ratio SILA trajectory.** Longitudinal biomarker trajectories against chronological age (**A**) and time from biomarker positivity (**B**) with each point representing a single timepoint, lines connecting points from the same participants, and Aβ+ participants shown in red with Aβ- shown in gray. Dashed lines indicate the amyloid positivity threshold. **B:** The SILA trajectory curve is shown. **C:** Estimated rate of change for a given p-tau_217_/Aβ_42_ value is shown with error shown by the shaded ribbon.


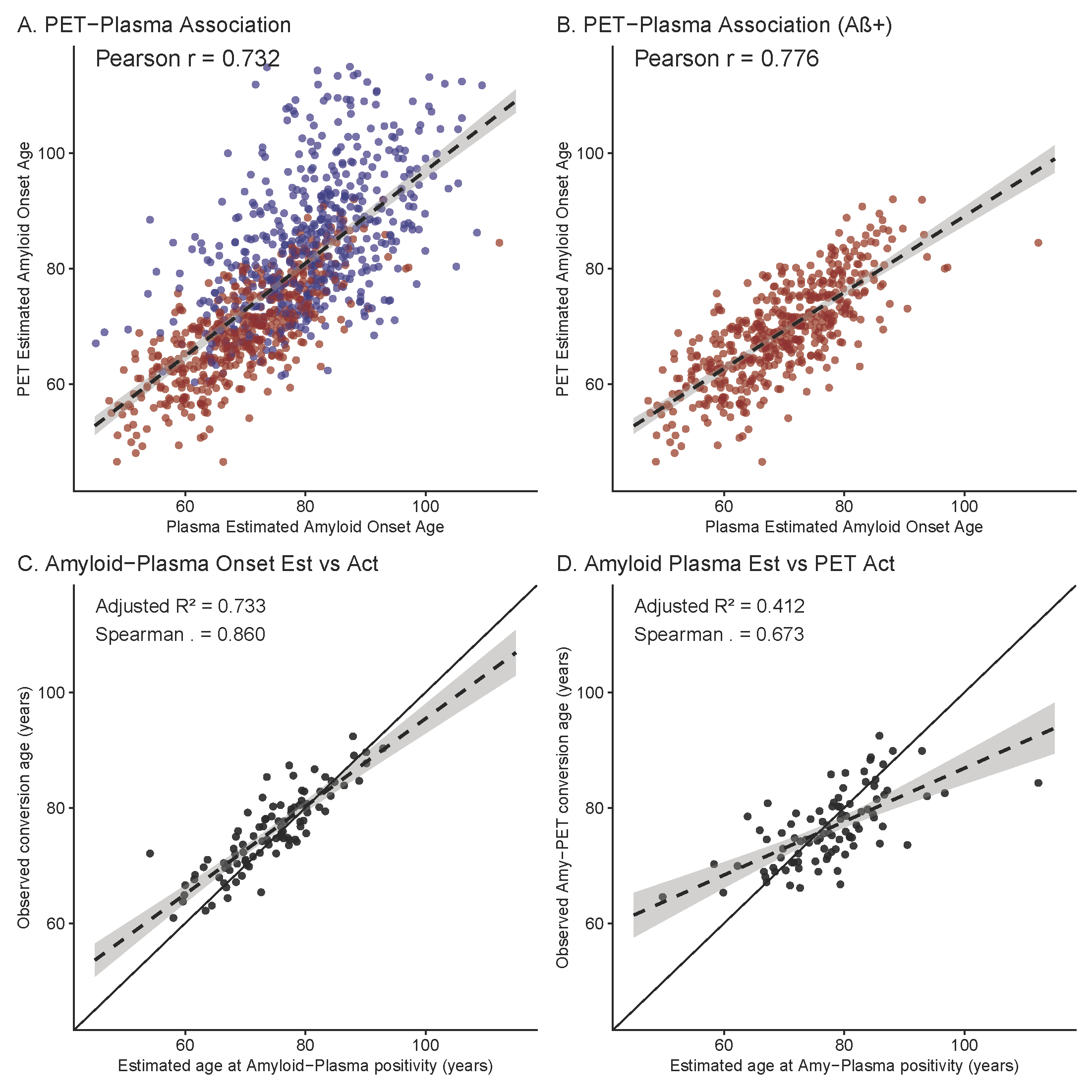


**Figure S3. Associations with Plasma p-tau_217_/Aβ_42_ estimated amyloid onset. A-B:** The association between plasma-estimated and PET-estimated amyloid onset age is shown in all (**A**) and Aβ+ (**B**) participants with Aβ+ individuals shown in red and Aβ- shown in blue. The dashed line represents the linear best-fit with the shaded ribbon indicating the 95% confidence interval for the linear fit. **C-D:** Association between estimated and actual onset age for within modality (**C**) and across modality with plasma estimated age predicting actual PET onset (**D**). The linear best fit is shown by the dashed line with the shaded ribbon showing the 95% confidence of fit, while the solid line represents the identity line for reference (slope = 1). The R^2^ and Spearman correlation are shown.


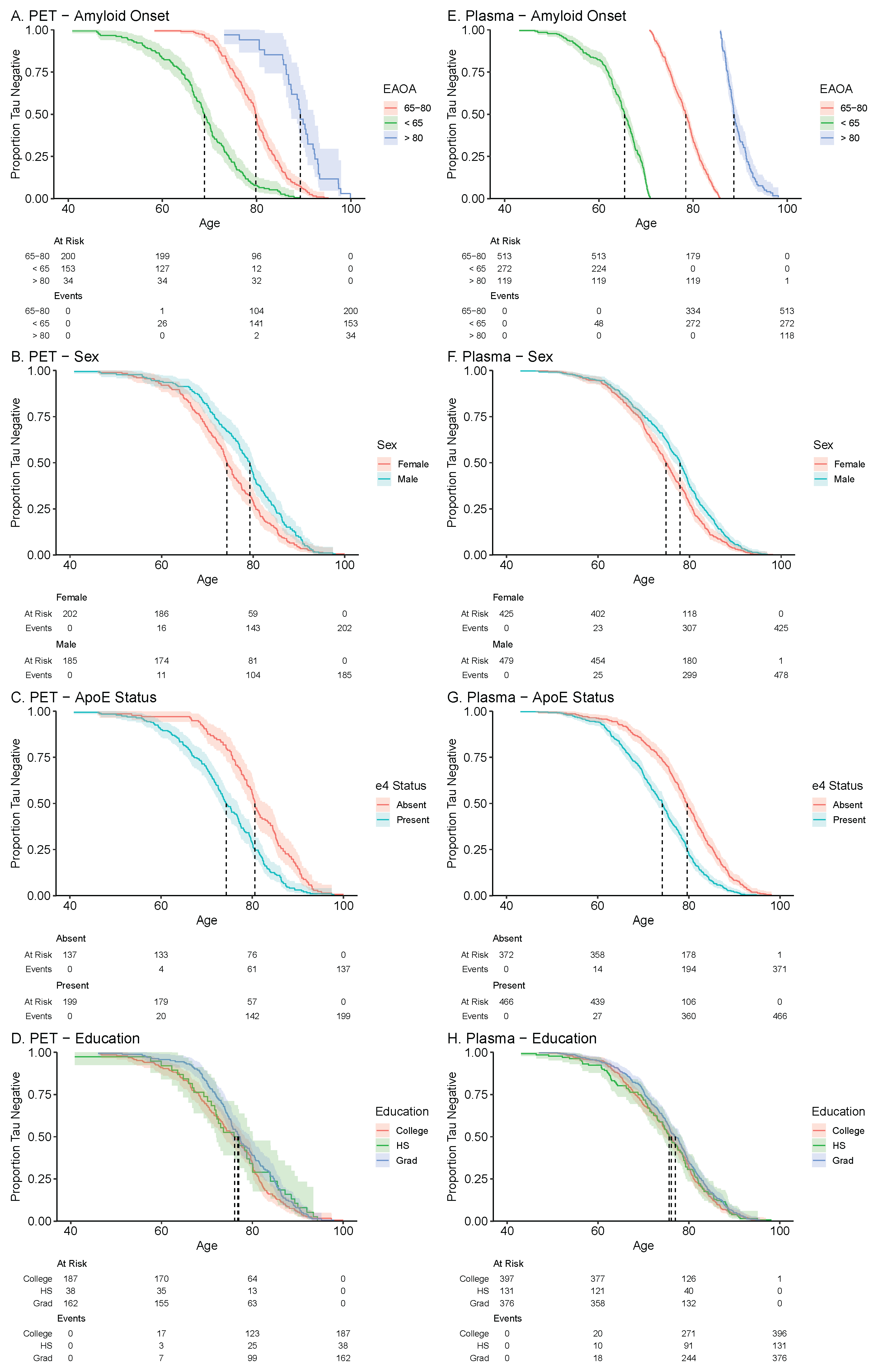


**Figure S4. Kaplan-Meier plots of univariable factors influencing estimated tau onset age.** Kaplan-Meier curves for the univariable cox-proportional hazard models for PET (**A-D**) and plasma (**E-H**) estimated tau onset age are shown. Lines indicate the survival curve and shaded ribbons reflect the 95% confidence interval. The dashed vertical lines indicate the age at which 50% of the individuals have had tau onset. Statistics for these models are reported in the main text in Figure 4. **A&E:** For models testing the impact of estimated amyloid onset age (EAOA), individuals were grouped for visualization.


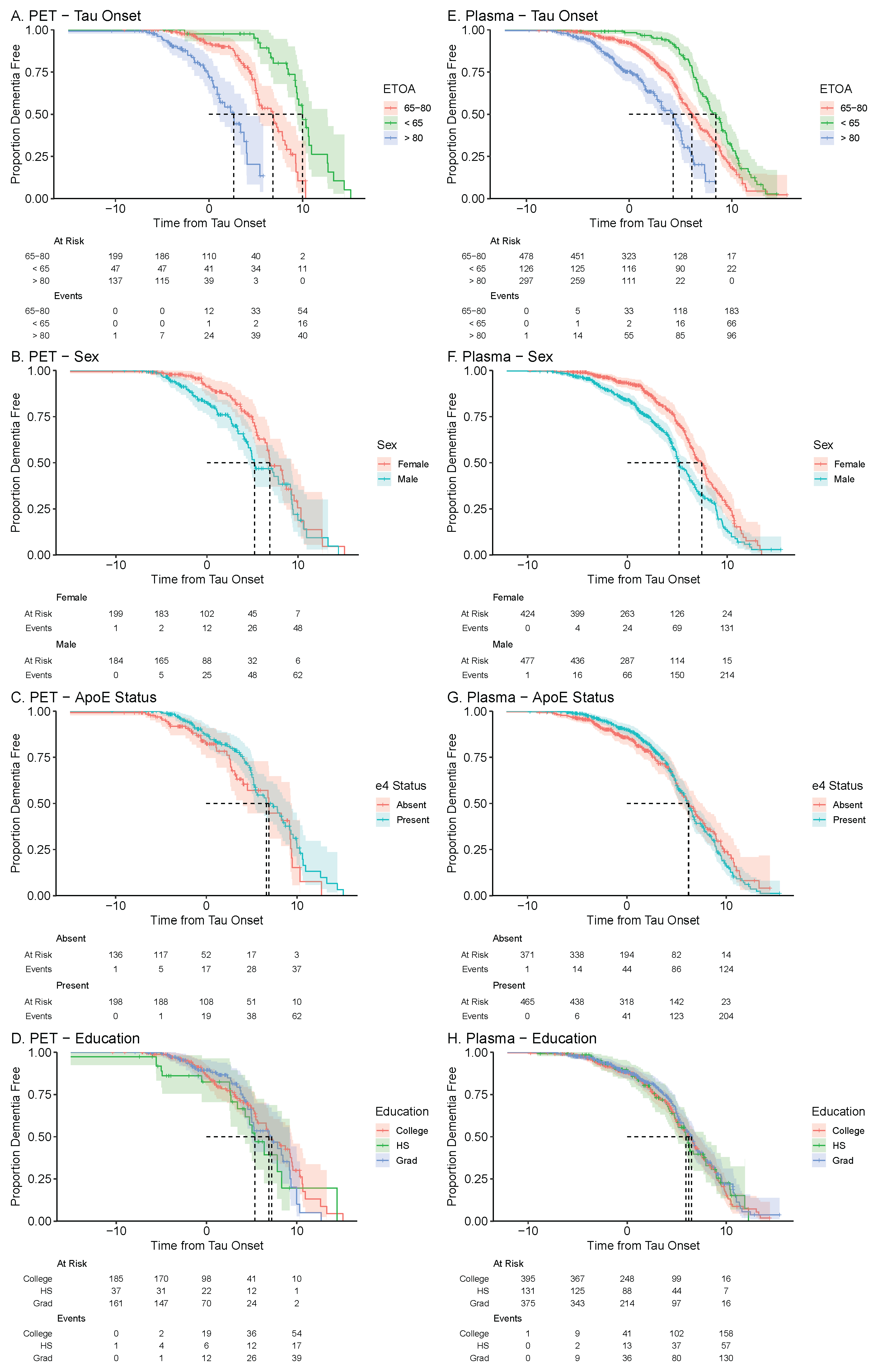


**Figure S5. Kaplan-Meier plots of univariable factors influencing time from tau onset to development of dementia.** Kaplan-Meier curves for the univariable cox-proportional hazard models for time from PET (**A-D**) and plasma (**E-H**) tau onset to dementia are shown. Lines indicate the survival curve and shaded ribbons reflect the 95% confidence interval. Censored data is depicted by vertical hashes on the survival curves. The dashed vertical lines indicate the age at which 50% of the individuals have a diagnosis of dementia. Statistics for these models are reported in the main text in Figure 5. **A&E:** For models testing the impact of estimated tau onset age (ETOA), individuals were grouped for visualization.

**Table S1. Correlation of estimated onset age with actual onset using different imputation methods**

|  | AFT | Midpoint | Left | Right | Random |
| --- | --- | --- | --- | --- | --- |
| Amyloid PET | *r* = 0.95 [0.93-0.97] | *r* = 0.95 [0.93-0.97] | *r* = 0.94 [0.92-0.96] | *r* = 0.95 [0.92-0.96] | *r* = 0.95 [0.92-0.96] |
| Amyloid Plasma | *r* = 0.93 [0.90-0.95] | *r* = 0.93 [0.90-0.95] | *r* = 0.91 [0.87-0.94] | *r* = 0.93 [0.90-0.95] | *r* = 0.91 [0.87-0.94] |
| Amyloid Plasma to PET | *r* = 0.73 [0.62-0.81] | *r* = 0.73 [0.62-0.81] | *r* = 0.73 [0.62-0.81] | *r* = 0.72 [0.61-0.81] | *r* = 0.74 [0.63-0.82] |
| Tau PET | *r* = 0.92 [0.84-0.96] | *r* = 0.96 [0.91-0.98] | *r* = 0.94 [0.88-0.97] | *r* = 0.96 [0.91-0.98] | *r* = 0.94 [0.88-0.97] |
| Tau Plasma | *r* = 0.95 [0.92-0.96] | *r* = 0.95 [0.92-0.97] | *r* = 0.93 [0.90-0.95] | *r* = 0.94 [0.92-0.96] | *r* = 0.93 [0.89-0.95] |
| Tau Plasma to PET | *r* = 0.75 [0.52-0.88] | *r* = 0.76 [0.53-0.88] | *r* = 0.75 [0.52-0.88] | *r* = 0.75 [0.53-0.88] | *r* = 0.73 [0.50-0.87] |

Pearson r [95% CI] for association between SILA-estimated onset age and actual onset age using various imputations for interval-censored actual onset age. AFT: Accelerated failure time using icenReg package; Midpoint: midpoint between last negative and first positive; Left: last negative time; Right: first positive time; Random: random time between last negative and last positive
